# Short-term transcriptomic analysis at organ scale reveals candidate genes involved in low N responses in NUE-contrasting tomato genotypes

**DOI:** 10.3389/fpls.2023.1125378

**Published:** 2023-03-03

**Authors:** Francesco Sunseri, Meriem Miyassa Aci, Antonio Mauceri, Ciro Caldiero, Guglielmo Puccio, Francesco Mercati, Maria Rosa Abenavoli

**Affiliations:** ^1^ Dipartimento Agraria, Università Mediterranea di Reggio Calabria, Reggio Calabria, Italy; ^2^ National Research Council of Italy, Institute of Biosciences and Bioresources (CNR-IBBR), Palermo, Italy; ^3^ Dipartimento di Scienze Agrarie, Alimentari e Forestali, Università degli Studi di Palermo, Viale delle Scienze, Palermo, Italy

**Keywords:** *Solanum lycopersicum* L., nitrogen use efficiency, abiotic stress, RNAseq, weighted gene co-expression network analysis (WGCNA)

## Abstract

**Background:**

Understanding the complex regulatory network underlying plant nitrogen (N) responses associated with high Nitrogen Use Efficiency (NUE) is one of the main challenges for sustainable cropping systems. Nitrate (NO_3_^-^), acting as both an N source and a signal molecule, provokes very fast transcriptome reprogramming, allowing plants to adapt to its availability. These changes are genotype- and tissue-specific; thus, the comparison between contrasting genotypes is crucial to uncovering high NUE mechanisms.

**Methods:**

Here, we compared, for the first time, the spatio-temporal transcriptome changes in both root and shoot of two NUE contrasting tomato genotypes, Regina Ostuni (high-NUE) and UC82 (low-NUE), in response to short-term (within 24 h) low (LN) and high (HN) NO_3_^-^ resupply.

**Results:**

Using time-series transcriptome data (0, 8, and 24 h), we identified 395 and 482 N-responsive genes differentially expressed (DEGs) between RO and UC82 in shoot and root, respectively. Protein kinase signaling plant hormone signal transduction, and phenylpropanoid biosynthesis were the main enriched metabolic pathways in shoot and root, respectively, and were upregulated in RO compared to UC82. Interestingly, several N transporters belonging to NRT and NPF families, such as *NRT2.3, NRT2.4, NPF1.2,* and *NPF8.3*, were found differentially expressed between RO and UC82 genotypes, which might explain the contrasting NUE performances. Transcription factors (TFs) belonging to several families, such as ERF, LOB, GLK, NFYB, ARF, Zinc-finger, and MYB, were differentially expressed between genotypes in response to LN. A complementary Weighted Gene Co-expression Network Analysis (WGCNA) allowed the identification of LN-responsive co-expression modules in RO shoot and root. The regulatory network analysis revealed candidate genes that might have key functions in short-term LN regulation. In particular, an asparagine synthetase (ASNS), a CBL-interacting serine/threonine-protein kinase 1 (*CIPK1*), a cytokinin riboside 5’-monophosphate phosphoribohydrolase (LOG8), a glycosyltransferase (*UGT73C4*), and an ERF2 were identified in the shoot, while an LRR receptor-like serine/threonine-protein kinase (*FEI1*) and two TFs *NF-YB5 *and *LOB37* were identified in the root.

**Discussion:**

Our results revealed potential candidate genes that independently and/or concurrently may regulate short-term low-N response, suggesting a key role played by cytokinin and ROS balancing in early LN regulation mechanisms adopted by the N-use efficient genotype RO.

## Introduction

Nitrogen (N) is an essential nutrient whose availability limits plant growth and development, causing crop yield and quality losses (Wang et al., 2018; Fredes et al., 2019). The extensive use of N fertilizers has been a strategy to boost agricultural production and meet global food demand (Guo et al., 2010). However, less than 50% of applied N is taken up by crops; the remaining is lost into the environment, causing pollution and, indirectly, damage to human health (Good and Beatty, 2011). Therefore, improving plant N use efficiency (NUE) is an effective and promising approach to reducing fertilizer use, maintaining crop yield, and alleviating detrimental impacts on the environment (Hu et al., 2018). Many efforts have been made to elucidate the complex regulatory networks underlying plant N responses as well as to identify N-responsive genes and transcription factors (TFs) associated with NUE (Han et al., 2016; Mauceri et al., 2021; Nazish et al., 2022). However, it is a very complex trait controlled by many factors, making the development of varieties with low N requirements difficult (Yadav et al., 2017).

Although nitrate (NO_3_^-^) and ammonium (NH_4_^+^) are the major inorganic N forms in aerobic agricultural soils (Wang et al., 2018), NO_3_^-^ is the most used by plants (Tischner, 2000; Crawford and Forde, 2002). Besides its role as a nutrient, NO_3_^-^ is a local and systemic signal molecule that coordinates many physiological processes essential for plant growth and development as well as its uptake (Alvarez et al., 2012; Ruffel et al., 2014). It also regulates the expression of genes involved in N assimilation and C metabolism (Scheible et al., 2004; Vidal and Gutiérrez, 2008), the root and shoot architecture, and delays flowering (Remans et al., 2006; Vidal et al., 2014; Yuan et al., 2016).

To orchestrate all these adaptive responses, NO_3_^-^ provokes plant transcriptome reprogramming (Canales et al., 2014; Medici and Krouk, 2014; Vidal et al., 2015; Mauceri et al., 2021), by which the transcript accumulation changes are cell- and tissue-specific, taking place very fast after NO_3_^-^ exposure (Krouk et al., 2010; Walker et al., 2017; Varala et al., 2018). Several transcription factors (TFs) belonging to different families, such as B-box containing proteins (BBXs), myeloblastosis (MYBs), ethylene response factors (ERFs), basic leucine zipper (bZIPs), NIN-like proteins (NLPs), lateral boundary domain-containing proteins (LBDs), BTB and TAZ domain proteins (BTs), have been recently identified as key regulator genes in the primary nitrate response (PNR) (Liu et al., 2017; Gaudinier et al., 2018; Wang et al., 2018; Brooks et al., 2019). Moreover, NO_3_^-^ induces dynamic changes in intracellular calcium signaling to generate rapid control of nitrate uptake and transcriptional PNR (Hu et al., 2009; Liu et al., 2017). Finally, it elicits post-translational modifications such as chromatin modification and protein phosphorylation and ubiquitination (Liu et al., 2017; Poza-Carrión and Paz-Ares, 2019), which can lead to rapid and reversible modifications that directly regulate the localization, stability, interaction, function, and enzymatic activity of target proteins (Yip Delormel and Boudsocq, 2019).

Recently, transcriptome analyses described different responses to limited N supply in crops such as wheat, rice, potato, and eggplant (Subudhi et al., 2020; Zhang et al., 2020; Zhang et al., 2021; Mauceri et al., 2021; Meng et al., 2021). In tomato, an array analysis of the root revealed N-induced genes that play a role in N nutrition, including transport and assimilation genes related to C and N metabolism as well as water channels, phosphate, and potassium transporters (Wang et al., 2001). More recently, an integrative transcriptomic and metabolomic approach was able to identify pathways and key regulatory genes in response to low N, again in tomato (Renau-Morata et al., 2021). Currently, a comparative transcriptome analysis of NUE contrasting genotypes in response to early LN resupply is not yet available.

Tomato (*Solanum lycopersicum* L.), as one of the most N-demanding crops to achieve optimal yields (up to 250–300 kg/ha) (Zotarelli et al., 2009), represents a reasonable target to develop sustainable tomato cropping systems. In this respect, the selection of high NUE tomatoes and the understanding of their responses to N-limited conditions are relevant. Recently, NUE-contrasting tomato genotypes have been identified among some long-storage ecotypes (Abenavoli et al., 2016; Lupini et al., 2017). Notably, the differences in NUE have been related to the ability to regulate long-distance N transport, assimilation, remobilization, and storage genes (Aci et al., 2021).

The present work aims to highlight the transcriptome modifications as well as the main metabolic pathways involved in the short-term LN-resupply responses in both root and shoot of high (Regina Ostuni, RO) and low (UC82) NUE tomato genotypes. This study allowed us to identify putative candidate genes and transcription factors regulating early LN-response useful for NUE improvement in tomatoes.

## Materials and methods

### Plant material and growth conditions

**Figure 1:**
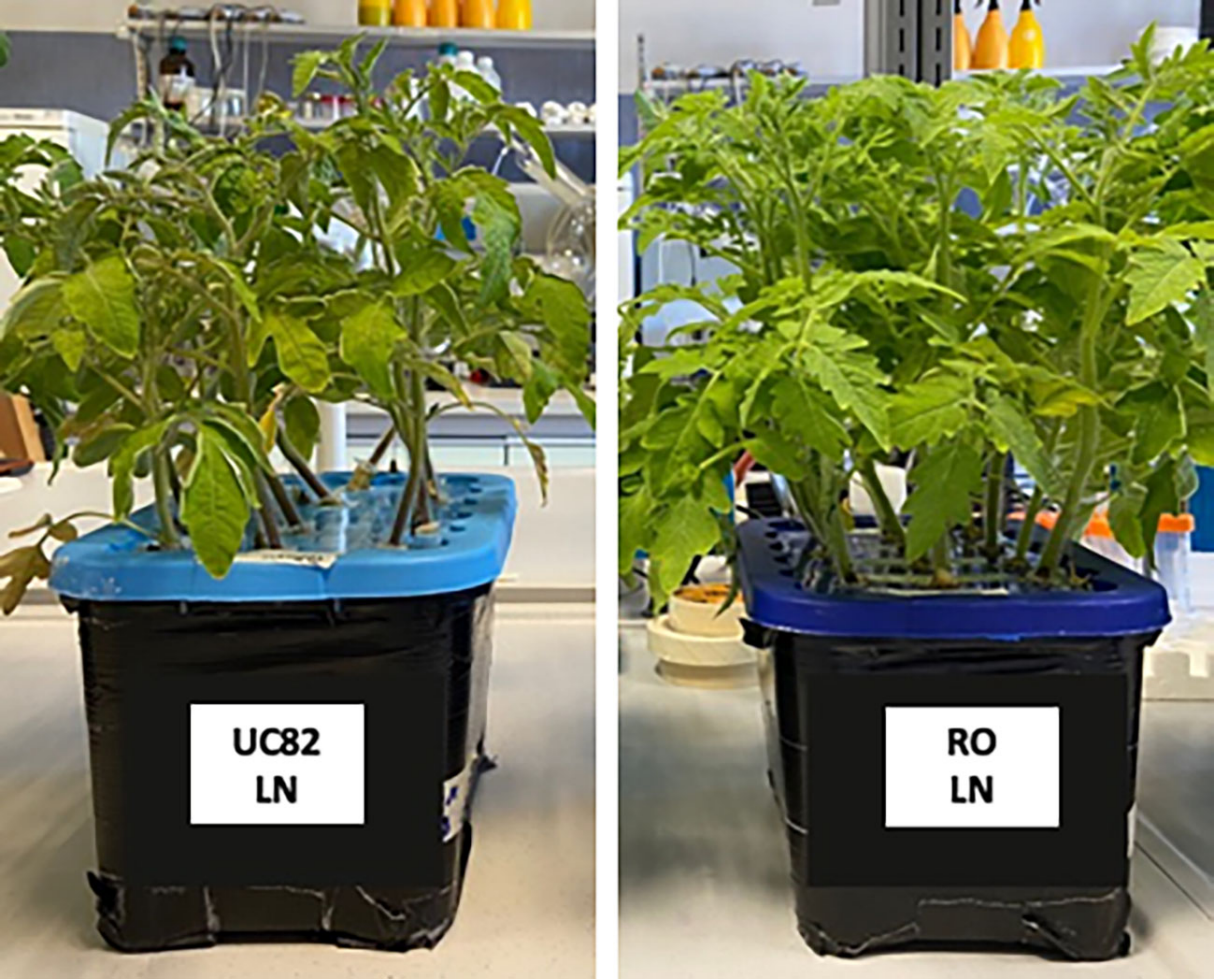
Tomato genotypes UC82 and Regina Ostuni (RO) grown in hydroponic systems at low N (LN) conditions.

Seeds of two NUE contrasting tomato genotypes, namely Regina Ostuni (RO) (high NUE) from Apulia (Italy) and UC82 (low NUE), a North American old cultivar from the University of California (Davis, USA), were sterilized with 10% (v/v) NaClO for 15 min, rinsed twice (**Figure 1**). Then, they are placed in magenta boxes containing 0.8% agarose gel (diluted in 0.5 mM CaSO_4_) for 10 days. Uniform selected seedlings were transferred into an aerated hydroponic system containing a complete Hoagland solution and grown for 10 days in a growth-controlled chamber (25°C, 70% RH, and 16 h photoperiod with a light intensity of 350 μmol m^−2^s^−1^) (Aci et al., 2021). The nutrient solution was renewed every 2 days, and the pH was maintained at 5.8 with 1M KOH. Since the internal NO_3_^-^ concentration influences the N regulatory mechanisms (Forde and Clarkson, 1999), a preliminary N-depletion experiment was carried out to confirm the time required for N starvation in both RO and UC82 as reported in Aci et al. (2021). The results confirmed that the best N-recovery starting point was set at 5 days from N-starvation in both tissues of each genotype. Therefore, RO and UC82 plants (20-d old), grown as reported above, were starved for 5 days and then resupplied with low (LN; 0.5 mM) and high (HN; 10 mM) NO_3_^-^ concentrations as previously established for tomato (Abenavoli et al., 2016; **Figure 1**). Both shoot and root of each genotype were harvested before N resupply (T_0_), at 8 h (T_1_), and 24 h (T_2_) after HN and LN resupply (**Figure S1**). Three biological replicates (a pool of three plants) were adopted for transcriptome analysis.

### RNA-seq analysis and data processing

Total RNA was extracted and purified using the RNeasy Plant Mini Kit (Qiagen, Milano, Italy), following the manufacturer’s protocol. RNA degradation and contamination were monitored on a 1% denaturing RNA agarose gel, and samples were quantified using a NanoDrop 2000 (ThermoFisher Scientific, Wilmington, Delaware, USA). cDNA libraries were constructed using 500 ng of total RNA for each genotype (RO and UC82), treatment (HN and LN), tissue (shoot and root), and time sampling (T_0_, T_1_, and T_2_) following the Transeq approach with single-end 60-bp reads as described by Tzfadia et al. (2018). The 60 libraries were sequenced on a six-lane HiSeq 2500 system (Illumina) using the SR60 protocol. The raw data were processed to obtain high-quality clean reads using BBDuk (http://jgi.doe.gov/data-and-tools/bb-tools/) to remove Illumina adapters and for quality trimming (k = 23, mink = 11, hdist = 1, trimq = 10, min length = 40). Clean reads were mapped to the *S. lycopersicum* (tomato) genome (SL3.0) from Ensemble Plants (http://plants.ensembl.org/Solanum_lycopersicum/Info/Index) using STAR v2.7.10 (Dobin et al., 2013). Principal component analysis (PCA) was carried out using PCAGO, an interactive web service useful for analyzing RNA-seq data to obtain a first characterization of biological sample clustering (Gerst and Hölzer, 2018).

### Differential gene expression analysis

Differentially Expressed Genes (DEGs) between treatments were detected using DESeq2 (Robinson and Oshlack, 2010; Love et al., 2014). Read counts were normalized using the size factor normalization method, and a Likelihood Ratio Test (LRT) was used to test multiple factors and their interactions (genotype, N-treatment, and sampling time). An adjusted p-value (Padj) of 0.05 was used as a threshold (Benjamini and Hochberg, 1995). Principal Component Analysis (PCA) and sample-to-sample distances were also evaluated using DESeq2. DEGs obtained from at least one comparison were used for the clustering phase through time-course analysis and co-expression network analysis. The tomato NPF gene supervised clustering was performed by the DEGreport R package (Pantano, 2019) using DESeq2 Variance Stabilizing Transformed (VST) data.

### Time-course analysis

To examine and visualize the DEG expression profiles and to identify N-responsive genes over time (0 h, 8 h, and 24 h) after N-resupply, the short time-series expression miner (STEM) software (Ernst and Bar-Joseph, 2006) was used. Each gene was assigned to the filtering criteria of the model profiles, and the correlation coefficient was determined. A standard hypothesis test using the true order of time-points, the number of genes assigned to the model profile, and the expected number of assigned genes was performed to detect significant enriched profiles for both genotypes (P-value ≤0.05, Bonferroni correction).

### Weighted gene co-expression network analysis

A Weighted Gene Co-expression Network Analysis (WGCNA) was performed on the DESeq2 variance stabilizing transformed (VST) expression data of the previously identified DEGs using the WGCNA package v1.51 (Langfelder and Horvath, 2008).

The analysis was performed on both tissues, distinctly. To select the soft threshold for both root and shoot analyses, the scale-free topology criterion was adopted (8 and 12, respectively). The adjacency matrix obtained from the correlation matrix of gene expression to construct the Topological Overlap Matrix (TOM) was used (Yip and Horvath, 2007). After hierarchical clustering, the highly correlated genes were assigned to the same module (Ravasz et al., 2002) through the Dynamic Tree Cut algorithm (minimum module size = 30). The similar modules were then merged into a single module using the correlation coefficients between their Module Eigengenes (ME) (the first principal component of the expression matrix) (threshold = 0.25). The module membership (MM) as well as the gene significance (GS) were calculated (Langfelder and Horvath, 2008). Finally, a network visualization and the selection of highly connected genes (hub-genes) were carried out by Cytoscape v3.8.2 (Shannon, 2003).

### RNA-seq data validation by RT-qPCR

To validate the transcriptomic results, RNA samples previously utilized for sequencing were used for quantitative real-time PCR (RT-qPCR) experiments on 10 key genes identified in both shoot and root. Total RNA was extracted and purified using a TRIzol™ reagent (Qiagen, Milano, Italy) according to the instructions provided by the manufacturer. The Maxima First Stand cDNA Synthesis Kit (Thermo Fisher Scientific Baltics UBA) was used to synthesize cDNA samples by RNA reverse transcription according to the manufacturer’s instructions. The primer specificity of candidate genes was verified by melting curves using the mixed cDNA as a template and 2% agarose gel electrophoresis analysis. The PowerUp SYBR Green master mix (Applied Biosystems by Thermo Fisher Scientific) and the Applied Biosystems QuantStudio™ 5 Real-Time PCR System were employed to perform qPCR with gene-specific primers designed using Primer3 (v0.4.0) and listed in **Table S1**. Three biological and three technical replicates were adopted, and the means of the relative gene expression (Ct) were normalized to the reference genes, Actin1 and Ef1-α (Løvdal and Lillo, 2009), and it was calculated for each gene by using the 2^−ΔΔCt^ method as described by Livak and Schmittgen (2001).

## Results

In the present study, RO and UC82 (high and low NUE tomato genotypes, respectively) transcriptomic profiles were analyzed. The experimental design included three time samplings (T_0_, before N resupply; T_1_ and T_2_, after 8 and 24 h N resupply, respectively) of both shoot and root, collected from plants resupplied with low (LN; 0.5 mM) and high (HN; 10 mM) NO_3_^-^.

### RNA-seq analysis

For this purpose, 60 cDNA libraries were constructed, generating 268 million clean reads, an expected number in agreement with the Transeq approach (Tzfadia et al., 2018), which were mapped to the tomato reference genome (SL3.0), yielding an overall mapping percentage of 72.14% (**Table S2**). After assembly, 35,845 transcripts were finally identified.

A principal component analysis (PCA) on the whole dataset showed high distinctiveness between time sampling in the shoot and in the root, with a clear distinction between shoot and root samples (**Figure S1**). Thus, the following analyses were distinctly performed on each tissue.

In all the possible combinations of G, N, and T factors, we identified 7,667 and 6,015 unique genes differentially expressed in shoot and root, respectively (*Padj<*0.05) (**Table S3**; **Figure S3**).

### Differentially expressed gene profiles trend during the time-course

**Figure 2:**
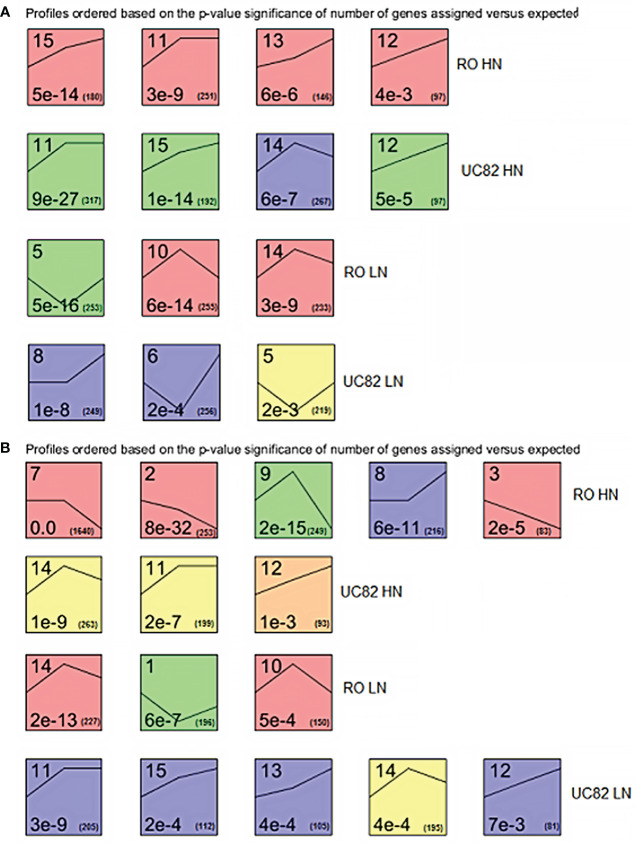
Significant enriched temporal expression profiles of the DEGs identified between genotypes, times, N levels, and their interactions in shoot **(A)** and root **(B)**. The number on the top refers to the cluster number. The numbers at the bottom are the *P*-values (left) and the gene number (right) assigned in each cluster, respectively. The lines inside each square represent the trend at the three experimental time points for each cluster.

DEG expression profiles across time were examined using STEM software to highlight clustered DEGs in shoot and root. The expression patterns of all the DEGs allowed us to identify 14 and 13 significant (*P*-value<0.05) different gene clusters in shoot and root, respectively, distinguished for each N condition and genotype (**Figure 2**). In the shoot, after HN resupply, four clusters were significantly enriched for both RO and UC82. A similar trend between genotypes (upregulation during the time) for the shared clusters (#11, 12, and 15) was highlighted, while cluster #13 was upregulated only in RO and #14 exhibited an initial increase followed by a decline for UC82. After LN resupply, three significant enriched clusters for both genotypes were detected, including an upregulated (#8) and two biphasic (with initial decrease followed by upregulation; #5, 6) clusters for UC82, and three biphasic ones with a contrasting trend (#5 *vs*. #10, 14) for RO (**Figure 2A**).

In the root, after HN resupply, five and three specific clusters were significantly enriched in RO and UC82, respectively. Three downregulated (#2, 3, and 7), one biphasic (#9), and one upregulated cluster belonged to RO, while two upregulated patterns (#11, 12) and one biphasic were found in UC82. After LN resupply, the same number of significant enriched clusters were detected but with an opposite profile between genotypes in comparison to HN: three in RO and five in UC82. In the latter genotype, four (#11, 12, 13, and 15) out of five clusters showed an upregulated pattern, while in UC82, all three clusters isolated (#1, 10, and 14) were biphasic (**Figure 2B**). Overall, DEG number increased across time (from 0 to 24 h) in both N conditions, except for the downregulated genes in the shoot, and was higher in the LN *vs*. HN in both tissues at 24 h.

### Gene ontology and KEGG enrichment analysis of DEGs after N-resupply

Functional characterization of up- and downregulated genes in the RO *vs.* UC82 comparison at each tissue, N supply (LN and HN), and time (at starvation, 8, and 24 h from N resupply), was performed using a GO term enrichment analysis for the main GO categories, Biological Process (BP), Molecular Function (MF), and Cellular Component (CC) (**Figures S4**, **S5**).

In the BP group, the upregulated genes were significantly enriched in the “oxidation–reduction process,” “response to stimulus,” “response to stress,” and “catabolic process” GO terms in both tissues (**Figures S4**, **S5**). Interestingly, 7.2% of the upregulated genes in shoot were included in “signaling” and “signal transduction” terms (**Figure S4**). In shoot, the downregulated genes were mainly included in the “protein metabolic process”, “response to stimulus”, “proteolysis”, and “transmembrane transport” GO terms (**Figure S4**), while “response to stimulus”, “localization”, and “transport” were the main GO terms enriched in root (**Figure S5**).

Concerning the MF categories, most of the upregulated genes in shoot were enriched in “hydrolase activity”, “catalytic activity acting on a protein”, and “oxidoreductase activity” categories (**Figure S4**), whereas “oxidoreductase activity”, “cation binding”, “metal ion binding”, and “cofactor binding” were significantly enriched in the root (**Figure S5**). The “cation binding,” “metal ion binding,” and “oxidoreductase activity” GO terms grouped the downregulated genes in shoot (**Figure S4**), while “hydrolase activity,” “transporter activity,” and “transmembrane transporter activity” were enriched in root (**Figure S5**).

Finally, in the CC categories, GO terms have not been found in the shoot, while “extracellular region” and “apoplast” were enriched in the root among the upregulated genes (**Figure S5**). Among the downregulated genes, more terms were identified in the shoot than the root, with “cell periphery”, “chloroplast”, “plasma membrane”, and “plastid” being the terms enriched in the shoot (**Figure S4**), “cell periphery”, “plasma membrane”, and “extracellular region” in the root (**Figure S5**). The Kyoto Encyclopedia of Genes and Genomes (KEGG) analysis did not show any significant enriched metabolic pathways for the downregulated DEGs in both tissues. By contrast, the upregulated genes were mainly enriched in “metabolic pathway” in both tissues, as well as “plant hormone signal transduction” and “MAPK (mitogen-activated protein kinases) signaling pathway” in the shoot and “biosynthesis of secondary metabolites” and “phenylpropanoid biosynthesis” in the root (**Figure S6**).

### DEG functional analysis in shoot

**Figure 3:**
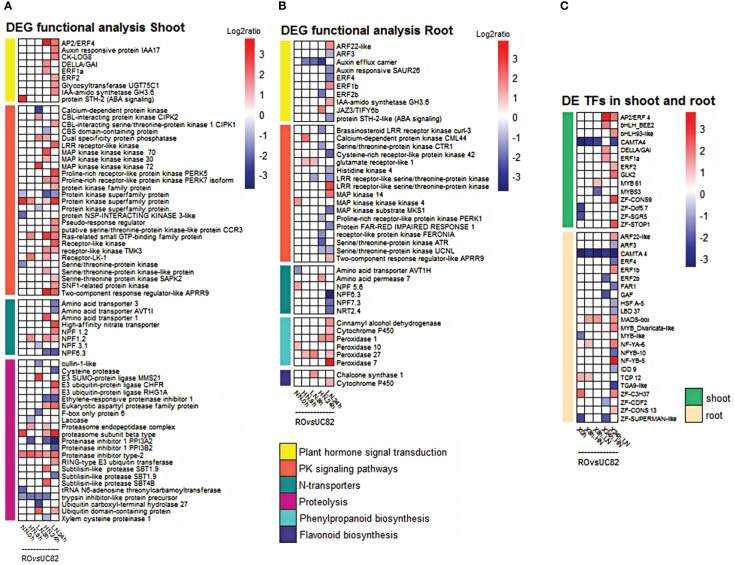
Expression level of DEGs included in functional classes based on GO term and KEGG pathway enrichment analyses in both tissues. Heatmap of DEGs involved in signal transduction, protein kinases signaling, N-transport, proteolysis, phenylpropanoid, and flavonoid biosynthesis in shoot **(A)** and root **(B)**, and the differentially expressed TFs in shoot and root **(C)** in the RO *vs*. UC82 comparison at 0, 8, and 24** h** after low (LN) and high (HN) nitrate resupply.

In response to the level of NO_3_^-^ resupplied, several hormone signaling-related genes were differentially expressed in shoots between genotypes. Two auxins (auxin-regulated *IAA17* and IAA-amido synthetase *GH3.6*), three ethylene response factors (*AP2/ERF4*, *ERF1a*, and *ERF2*), a cytokinin activating enzyme (cytokinin riboside 5’-monophosphate phosphoribohydrolase, LOG8) and a gibberellic acid signaling (DELLA-GAI) related genes, resulted more expressed in RO compared to UC82, after 24 h LN resupply (**Figure 3A**). Many protein kinases (PKs), which act as signal transducers or receptor proteins in protein phosphorylation, were also found differentially expressed (DE) between the two genotypes in both tissues, mainly at 24 h after LN resupply. In particular, 22 PKs were identified, including four receptor-like protein kinases (RLKs), four serine/threonine protein kinases (STPKs), four protein kinases family proteins, three mitogen-activated protein kinase (MAPKKKs), two CBL-interacting protein kinase kinase kinase (CIPK), two receptor-like protein kinases (RPKs), as well as a SNF1-related protein kinase, a protein NSP-interacting kinase 3-like, and a calcium dependent protein kinase (CDPK) (**Figure 3A**). Among the PKs, a *CIPK2* and *MAPK72* as well as a CDPK and a PK superfamily protein resulted up- and downregulated, respectively, after 8 h LN resupply, while two RLKs, two STPK, a RPK, a CIPK, and a SNF1-related protein kinase appeared upregulated at the same condition (LN) but at 24 h in RO compared to UC82 (**Figure 3A**).

In the early response to LN resupply, many DEGs identified in shoots were enriched in proteolysis (protein degradation processes), which regulates the availability of organic N for remobilization and allocation to N-demanding tissues. Genes encoding proteasome subunits and E3 ubiquitin-protein ligase, involved in the ubiquitin-proteasome system, appeared more expressed in RO compared to UC82. In particular, the proteasome subunit beta type and an E3 ubiquitin transferase transcript were more abundant in RO at 24 h in LN compared to HN condition, as well as an E3 SUMO-protein ligase *MMS21* and two E3 ubiquitin-protein ligase (*CHFR* and *RHG1A*) encoding genes, which were upregulated after 8 and 24 h LN resupply, respectively (**Figure 3A**). Moreover, many protease/proteinase inhibitor related genes were downregulated in RO compared to UC82 only after 24 h LN resupply, among which are an ethylene-responsive proteinase inhibitor, a proteinase inhibitor 1 *PPI3A2*, and a proteinase inhibitor 1 *PPI3B2* (**Figure 3A**).

### DEG functional analysis in root

In the root, many DEGs were involved in plant hormone signal transduction and the PK signaling pathway in the RO *vs*. UC82. Five auxins (an auxin-responsive protein *SAUR26*, an auxin efflux carrier, two-auxin response factor *ARF*, and the IAA-amido synthetase *GH3.6*), an abscisic acid (ABA) (protein *STH-2-like*), a jasmonic acid (*JAZ3/TIFY6B*), and a brassinosteroid (*CURL3*) related genes, as well as three ethylene-responsive transcription factors (*ERF4*, *ERF1b*, and *ERF2b*), involved in plant hormone signal transduction, were identified (**Figure 3B**). In detail, *GH3.6*, *ARF22-like*, and *ERF1b* were upregulated, while *SAUR26*, *ARF3*, *ERF4*, and *STH-2-like* were downregulated in the N-use efficient genotype (RO) after 24 h LN resupply (**Figure 3B**). Besides, fifteen PKs were identified, among which three STPKs, three MAPKs, three RPKs, three receptor-like kinases (LRR-RLK), as well as a histidine kinase (HK4), a CDPK, and a protein kinase domain (**Figure 3B**). *MAPK14* and *LRR-RLK* were upregulated, while *HK4*, *MKS1*, and a cysteine-rich RPK42 were downregulated in RO *vs*. UC82 after 24 h LN resupply (**Figure 3B**).

Finally, several genes involved in the phenylpropanoid and flavonoid pathways, including a chalcone synthase (*CHS1*) (after 8 h), a cinnamyl alcohol dehydrogenase (*CAD*), a cytochrome P450, and the peroxidase 7, were upregulated in root RO at the 24 h LN condition (**Figure 3B**).

### Differentially expressed transcription factors after N-resupply

In the shoot, 14 TFs, including an ethylene response factor (*AP2/ERF4*), two basic helix-loop-helix (*bHLH*), a calmodulin-binding transcription activator (*CAMTA4*), a Gibberellic Acid Insensitive (*DELLA/GAI*), two ethylene responsive transcription factors (*ERF*), a Golden2-like 2 (*GLK2*), two MYBs, and four zinc finger proteins (*ZF*) were differentially expressed between genotypes after 8 and 24 h LN or HN resupply (**Figure 3B**).

In the root, 23 differentially expressed TFs between genotypes were identified. Two-auxin response factor (*ARF*), a *CAMTA4*, three *ERF*, a protein far-red impaired response 1 (*FAR1*), a GAGA-binding transcriptional activator (*GAF*), a heat stress transcription factor A-5 *(HSF A-5*), a lateral organ boundaries (*LOB/LBD37*), a MADS-box transcription factor, two MYB, three nuclear factors Y (*NFYA6*, *B5*, and *B10*), a protein indeterminate-domain (*IDD9*), a Teosinte branched1/Cincinnata/Proliferating cell factor (*TCP*), a TGACG motif-binding protein (*TGA*), and four ZF proteins (**Figure 3C**). All the differentially expressed TFs were tissue-specific, except *CAMTA4*, which appeared downregulated in RO *vs*. UC82 in both tissues regardless of time sampling and N-condition. Overall, 50% and 56.5% of TFs identified were differentially expressed at LN condition in shoot and root, respectively, mainly after 24 h. In shoot, *bHLH93-like*, *ERF2*, *GLK2*, *ZF-Constans-9-like*, and *ZF-STOP1* were upregulated in RO after 24 h LN resupply. In root, 17 out of 23 TFs exhibited differential expression between RO and UC82 after 24 h LN resupply, of which eight TFs resulted upregulated in RO (**Figure 3C**).

### Weighted gene co-expression network analysis

**Figure 4:**
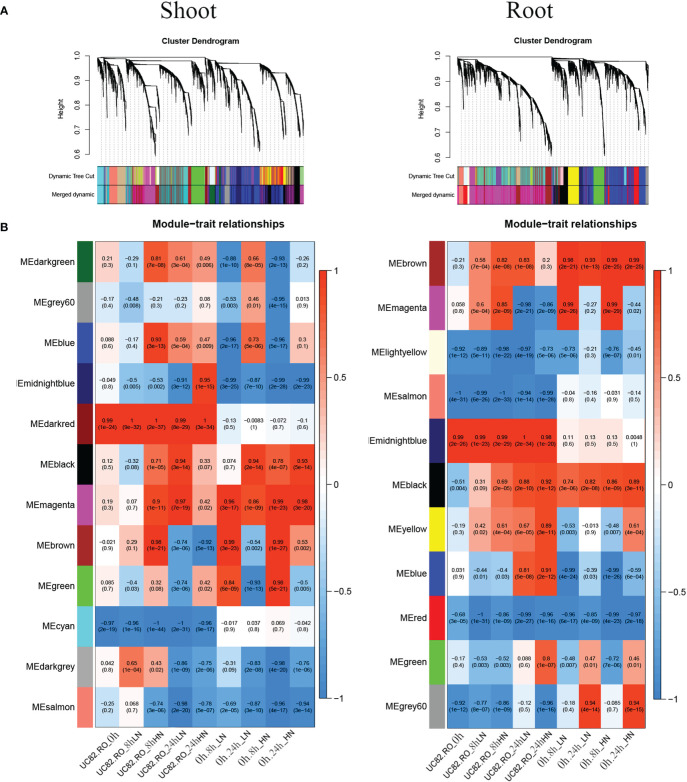
Merged clusters and dendrograms **(A)** and module–trait relationships **(B)** were obtained through the WGCNA analysis using 7,667 and 6,015 DEGs identified in shoot and root, respectively. In the heatmap, each Module Eigengene (ME) was correlated to each experimental condition. Inside each condition (0 h, 8 h-LN, 8 h-HN, 24 h-LN, 24 h-HN), the two genotypes were coded as RO (0) and UC82 (1) (columns 1–5), and ME were also correlated to each experimental condition regardless of the genotype (columns 6–9).

To identify the co-expression modules correlated to short-term N resupply and the hub genes involved in their transcriptional regulatory networks by using the NUE contrasting genotypes, a weighted gene co-expression network analysis (WGCNA) was carried out, including 7,667 and 6,015 DEGs identified in shoot and root, respectively, by using DESeq2. Our results revealed 11 co-expressed modules in the root and 12 in the shoot, gathering from 62 to 1,779 and from 49 to 1,979 genes, respectively (**Figure 4A**; **Figure S7**). Module Eigengenes (ME) were used to evaluate the Pearson correlation coefficient between each module and sample condition (**Figure 4B**); a box plot indicated the time course expression level for the midnight blue and magenta modules in shoot and root, respectively (**Figure S8**).

The dark red, green, and cyan modules in the shoot, as well as the midnight blue, yellow, and salmon modules in the root, exhibited limited differences in expression levels regardless of N levels (LN and HN) and time of sampling (0 h, 8 h, and 24 h). The N-responsive modules showed similar trends with significant changes in expression levels in both genotypes. In particular, the midnight blue, salmon, and magenta modules in the shoot and the gray60, blue, magenta, and black modules in the root (**Figure 4B**) exhibited the highest differences between genotypes. The N-responsive modules also showed four types of induction, which confirmed the evidence from the STEM analysis. In particular, the most abundant modules, magenta in the root and brown in the shoot, were quickly induced at 8 h after N-resupply for both treatments (LN and HN). The blue modules in both tissues showed a biphasic expression pattern, with a fast downregulation at 8 h and an upregulation at 24 h, while the gray60 (root) and the black (shoot) modules were upregulated mainly at 24 h for both N-resupply. Finally, the black (root) and magenta (shoot) modules showed a steady increase in upregulation after LN-HN resupply.

**Figure 5:**
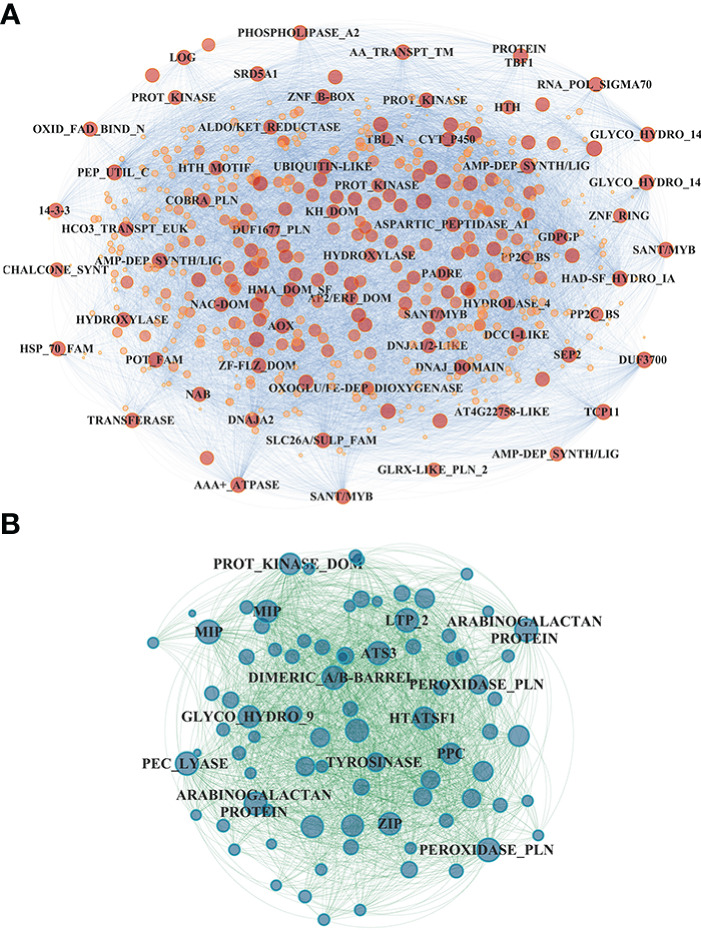
Network visualization of the midnight blue module detected in the shoot **(A)** and the gray60 module detected in the roots **(B)**. Hub gene annotation (SL3.2) is highlighted on each node.

In the shoot, the midnight blue and salmon modules, highly downregulated at both sampling times and N treatments, grouped 586 and 625 genes, respectively (**Table S4**); in particular, the salmon module showed a significant lower downregulation in RO compared to UC82. The magenta module (918 genes) resulted in high upregulation by N (LN and HN) in RO at both 8 and 24 h, while the brown module (1,979 genes) showed a significant upregulation induced by N in RO after 8 h and a strong downregulation after 24 h N resupply. GO enrichment analysis of the midnight blue and salmon modules showed a significant enrichment in water and inorganic molecule transporter activity, ubiquitination, and oxidoreduction processes. The magenta and brown modules were enriched in translation regulation processes, mRNA and DNA binding, N-methyltransferase activity, and RNA-binding molecular functions GO terms (**Table S5**). In the shoot, 72 hub genes in the midnight blue module were identified (**Figure 5A**; **Table S6**). Among these, an asparagine synthase (*ASNS*, Solyc01g079880.3), a CBL-interacting serine/threonine-protein kinase 1 (*CIPK1*, Solyc05g053210.3), a cytokinin riboside 5’-monophosphate phosphoribohydrolase (*LOG8*, Solyc06g075090.3), a glycosyltransferase (*UGT73C4*, Solyc10g085870.1), a sulfate transporter 3.1 (*SULTR3.1*, Solyc09g082550.3), an alternative oxidase 1 (*AOX1*, Solyc08g075540.3), and an ethylene-responsive transcription factor 2 (*ERF2*, Solyc01g090340.2) were identified (**Table S7**).

In the root, the gray60 (including 84 genes), black (395), and magenta (1,779) modules appeared highly induced by N resupply. The gray60 module was significantly upregulated after 24 h from N resupply in RO, while the black module showed a higher induction in UC82 at both 8 h and 24 h. Furthermore, the magenta module, including the highest gene number, showed a rapid induction at 8 h with a rapid downregulation after 24 h, more evident in the UC82 genotype. The gray60 module was significantly enriched in passive transmembrane transporter activity, channel activity, water transport, and zinc ion transport molecular function GO terms (**Table S5**). Moreover, 17 hub genes mainly involved in water and zinc ion transmembrane transport and oxidoreductase activity, including two aquaporins (Solyc10g055630, Solyc12g044330), two peroxidases (Solyc02g064970, Solyc02g084800), and two protein kinases (Solyc09g008860, Solyc10g007290) were detected in the gray60 module (**Figure 5B**, **Table S7**). The magenta module was significantly enriched in amino acid N-methyltransferase activity and translation regulatory processes GO terms (**Table S6**). Among the 270 hub-genes identified in the magenta regulatory network, we identified a high-affinity nitrate transporter (*NRT2.4*, Solyc11g069750), a low-affinity nitrate transporter (*NPF7*, Solyc04g079530), an LRR receptor-like serine/threonine-protein kinase (*FEI1*, Solyc01g109650.3), a style cell-cycle inhibitor 1 (*SCI1*, Solyc05g008750.3), two *MYBs*, an *AP2/ERF* transcription factor (Solyc06g076350, Solyc06g053610, Solyc06g063070), and a translation initiation factor *IF2/IF5* (Solyc06g082580) (**Table S7**). The blue module showed an opposite trend compared to the magenta module, being downregulated after 8 h of N resupply in both genotypes and significantly more expressed in UC82 after 24 h. It was mainly enriched in water and passive transmembrane transport activity and hydrolase activity (**Table S6**). By contrast, the black module grouped genes upregulated by N resupply with increasing expression levels across time, mainly in UC82. They were involved in nitrate and inorganic transmembrane transport, peroxidase, and oxidoreductase activity, and ribosomal constituents.

### N transporter modulated expression during short-term N resupply

Several transporters involved in both high- and low-affinity NO_3_^-^ systems were differentially expressed in both tissues and genotypes. In particular, the *NRT2.3* (Solyc06g074990) and the *NRT2.4* (Solyc11g069750), two high-affinity NO_3_^-^ transporters, were found to be differentially expressed. In UC82, the *NRT2.3* was downregulated in shoot, especially at 24 h, while the *NRT2.4* was highly upregulated in root, mainly at 8 h in both LN and HN.

In the nitrate/peptide transporter family (NPF), 30 and 23 members were detected among DEGs in shoot and root, respectively, of which seven were shared between tissues. In the shoot, 10 of 30 *NPFs* clustered in the N responsive modules, of which six were included in the midnight blue module (**Table S4**). In the root, 16 of 23 NPF members clustered in the N responsive modules, of which 11 were included in the blue module (**Table S5**).

**Figure 6:**
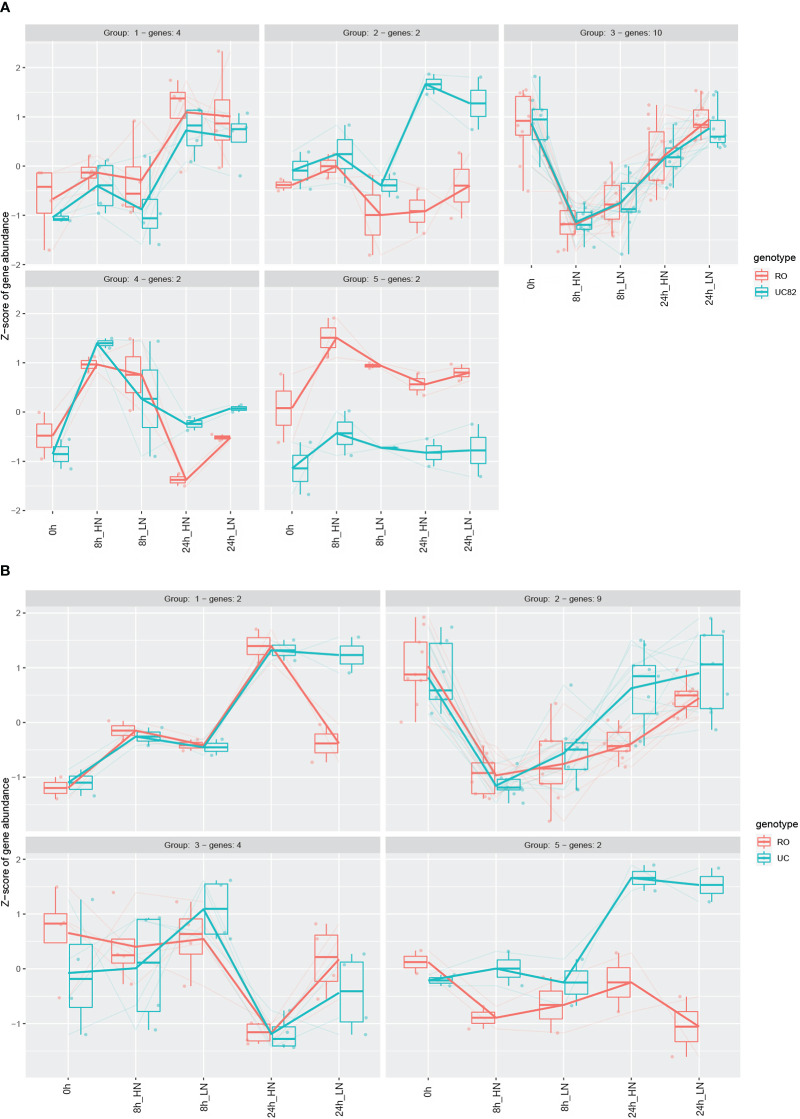
Expression profiles of the differentially expressed NPF genes in shoot **(A)** and root **(B)**. Genes were clustered through the DEGreport R package using Variance Stabilized Transformed (VST) data.

The NPF members differentially expressed were then clustered by using the Degprofiler package (**Figure 6**, **Table S8**), and five and four clusters were detected in the shoot and root, respectively. Groups 3 (shoot) and 2 (root) included a higher number of genes (**Figures 6A, B**), which were strongly downregulated after N resupply, especially at HN compared to LN, and this N treatment effect was more marked in the shoot than in the root. After 24 h, a significant re-induction (upregulation) was observed in both groups. Furthermore, in group 2, the *NPF6.3* (Solyc08g007430) and another *NPF1.2* isoform (Solyc05g006000) transporters resulted in upregulation of UC82 at 24 h in both N conditions (**Figure 6A**). More interestingly, group 5 that includes the *NPF1.2* (Solyc12g044310) and *NPF8.3* (Solyc12g042250) transporters showed a higher upregulation in RO compared to UC82 in shoot (**Figure 6A**). Finally, group 4, including two isoforms of *NPF7.3* (Solyc01g080870 and Solyc10g024490), was significantly upregulated in UC82 compared to RO in root, mainly after 24 h of both LN and HN resupply (**Figure 6B**).

### RNA-seq data validation by RT-qPCR

To validate the accuracy of the RNA-seq expression patterns, 10 key candidate genes, five from the shoot (*ASNS*, *CIPK1*, *LOG8*, *GLK2*, and *ERF2*) and five from the root (*AnnSl5*, *FEI1*, *NF-YB5*, and *LOB37*) for RT-qPCR validation, were chosen. The results were plotted in a scatter plot, revealing that the relative expressions of these genes were significantly in agreement with RNA-seq data, as reflected by a high correlation coefficient (R^2^ = 0.94, *P<*0.0001) between the methods (**Figure S9**).

## Discussion

Improving nitrogen use efficiency (NUE) in crops has become a significant challenge for sustainable agriculture. To achieve this goal, a deep understanding of plant responses to variable soil N availability, at both physiological and molecular levels is crucial. This knowledge is critical to dissecting the regulatory networks of this complex trait. The transcriptomic approach contributed to understanding the changes regulating crop responses to abiotic stress such as low N availability as well as to identifying key genes related to N-stress tolerance comparing NUE-contrasting genotypes (Goel et al., 2018; Sinha et al., 2018; Subudhi et al., 2020; Sultana et al., 2020; Mauceri et al., 2021; Puccio et al., 2022). In tomato, transcriptomic analyses led to the identification of genes differentially regulated by N availability (Wang et al., 2001; Renau-Morata et al., 2021), while no comparative transcriptome profiling between NUE-contrasting genotypes in response to N starvation and resupply has been reported so far.

In this study, the early molecular responses to low NO_3_^-^ (LN) at tissue scale in two NUE contrasting tomato genotypes, RO (N-use efficient) and UC82 (N-use inefficient) (Abenavoli et al., 2016; Aci et al., 2021), were evaluated. The tissue-specific transcriptome time-course analysis revealed the highest LN-sensitivity of the root compared to the shoot, in contrast with other findings revealing the shoot as the more responsible tissue for low N (Renau-Morata et al., 2021). These results were confirmed by the number of N-responsive genes (3,000 *vs*. 2,000), among which 482 and 395 were differentially expressed between genotypes in root and shoot, respectively (**Table S3**). Similar responses to LN were already observed in potato (Tiwari et al., 2020) and spinach (Joshi et al., 2020), confirming the central role played by root in NUE. Interestingly, our experimental design, in which two NUE contrasting genotypes were included, allowed us to identify DEGs, hub genes, and a network of co-expressed genes between the N-use efficient genotype, RO, and the N-use inefficient genotype, UC82.

### LN resupply promotes a differential spatio-temporal genes expression in NUE-contrasting genotypes

The N-responsive DEGs in the RO *vs*. UC82 showed significant differences between LN and HN in both tissues and across times of sampling, mainly after 24 h. Our results suggested that these DEGs, in the early response to LN resupply, might govern NUE performances in tomatoes. The GO and KEGG pathways enrichment analyses identified tissue-specific biological processes and pathways related to “plant hormone signal transduction” and “protein kinase signaling” as well as “phenylpropanoid and flavonoid biosynthesis” pathways significantly enriched in the shoot and root in the high NUE genotype (RO), respectively (**Figures S4**, **S5**). Thus, for their key role in NO_3_^-^ signaling and stress adaptation, we focused our attention on genes belonging to these last pathways. Among the DEGs included in “plant hormone signal transduction,” two and five auxin-related genes were identified in shoot and root, respectively. In detail, an IAA amido-synthetase *GH3.6*, which regulates auxin excess in plants (Nakazawa et al., 2001; Staswick et al., 2005), was upregulated in RO in both the shoot and root, suggesting a synergic regulation of shoot and root auxin content in the N-use efficient genotype during early LN response (**Figure 3**). Auxin transport and signaling play a critical role in plant adaptation to N availability (Krouk et al., 2010; Vanstraelen and Benková, 2012; Wang et al., 2019), which in turn significantly alters auxin biosynthesis, transport, and transduction (Asim et al., 2020). Moreover, nitrate and auxin signaling might overlap in root system architecture regulation (Walch-Liu and Forde, 2008; Asim et al., 2020). In addition, two auxin response factor genes (*SAUR26* and *ARF3*) were also downregulated in RO compared to UC82 in the root after 24 h LN resupply. The ARF family was involved in the response to LN supply, as previously reported. For example, *ARF18*, an auxin response factor, regulates *NRT2.4*, *DUR3*, and *AMT1.2* expression in *Arabidopsis* and tomato (Gaudinier et al., 2018; Renau-Morata et al., 2021).

Furthermore, putative tomato cytokinin riboside 5’-monophosphate phosphoribohydrolase *LOG8* transcripts, the main enzyme converting inactive cytokinin nucleotides to the biologically active free-base form (Kuroha et al., 2009), were more abundant in the RO shoot compared to UC82 after 24 h LN resupply. Cytokinins are signaling molecules that indicate plant N status (Sakakibara, 2006; Sakakibara, 2021). Besides their role in root–shoot–root communication (Narcy et al., 2013; Naulin et al., 2020), they can repress high-affinity NO_3_^-^ transporter genes (Ruffel et al., 2011), as well as induce N-metabolism-related genes such as nitrate reductase (*NR*) (Gaudinova, 1990). Accordingly, our results confirmed a potential crosstalk between NO_3_^-^ and cytokinin signaling in tomatoes. Finally, many ethylene responsive transcription factors (ERFs) genes, such as *AP2/ERF4* and *ERF2* in shoot as well as *ERF1b* in root, resulted in RO upregulation after 24 h LN resupply. *ERFs* are involved in ethylene signaling pathways and regulate many stress-related gene expressions controlling plant growth and development (Kazan, 2015; Xiao et al., 2016). Similar results were already observed in contrasting N-responsive genotypes of rice, barley, and spinach in response to LN (Quan et al., 2016; Xie et al., 2019; Joshi et al., 2020; Sun et al., 2020).

The mitogen-activated PKs (*MAPKs*, *MAPKKs*, and *MAPKKKs*) in the “protein kinase signaling” pathway are reported to be involved in plant stress resistance signal transduction, NO_3_^-^ sensing, and metabolism in several plants (Hu et al., 2009; Hao et al., 2011). *MPK7* is responsible in *Arabidopsis* for phosphorylating the nitrate reductase (*NR2*) and LOB domain binding proteins (LDB37 and LDB39), involved in NO_3_^-^ signaling and targeted by many *MAPKs* (Chardin et al., 2017). Five *MAPKKKs* were identified as direct targets of the NIN Like Protein 7 (*NLP7*) TF, a master regulator of early nitrate signaling in the root (Marchive et al., 2013; Chardin et al., 2017). In agreement, we also identified three *MAPKKKs* and *MAPK14* upregulated in the shoot and root, respectively, in the RO *vs*. UC82.

Moreover, some leucine-rich repeat receptor-like kinases (*LRR-RLKs*) were more expressed in the N-use efficient genotype (RO) in both tissues. The *LRR-RLKs* are involved in many critical biological processes, including growth, development, and abiotic stress responses (de Lorenzo et al., 2009). Several genes encoding different subfamilies of *RLKs* are regulated by NO_3_^-^, but these responses appear sometimes contrasting depending on cell types, organs, developmental stages, and growth conditions (Liu et al., 2020). In our experiment, the *LRR-RLK*s appeared involved in tomato response to low N. In addition, two CBL-interacting protein kinases (*CIPK1* and *CIPK2*) were upregulated in the shoot of RO compared to UC82 after 8 and 24 h LN resupply. By contrast, Ca^2+^-dependent PKs (*CDPK*) were strongly downregulated after 8 h of LN resupply. These *PKs* are involved in the regulation of the cross-link between Ca^2+^ and NO_3_^-^ signaling and uptake regulation (Sakakibara, 2003; Hu et al., 2009). More interestingly, recent studies revealed that NO_3_^-^ resupply stimulated rapid CIPK2 phosphorylation, underlining the important role of NO_3_^-^-activated Ca^2+^-sensor protein kinases (*CPKs*) and the NO_3_^-^–CPK–NLP regulatory network (Liu et al., 2017; Liu et al., 2020).

Finally, the limited NO_3_^-^ availability induces “phenylpropanoids and flavonoid biosynthesis,” which represents a plant adaptive strategy to LN stress (Diaz et al., 2006; Peng et al., 2008). Interestingly, the high-NUE genotype RO displayed in the root higher transcriptional levels of genes related to the phenylpropanoid and flavonoid biosynthesis pathways compared to UC82 after LN resupply. A chalcone synthase 1 (*CHS1*) and a cinnamyl alcohol dehydrogenase (*CAD*) gene, key enzymes in flavonoids, anthocyanins, and other phenylpropanoid compound biosynthesis (Tobias and Chow, 2005; Dao et al., 2011), resulted in RO upregulation after 8 h and 24 h LN resupply, respectively. The upregulation of genes involved in the phenylpropanoid pathway and others encoding flavonoids, described as signal molecules in root-to-shoot signal transduction in plants exposed to N deficiency, was frequently underlined (Buer et al., 2007; Brunetti et al., 2013; Quan et al., 2016; Goel et al., 2018; Sun et al., 2020).

### N transporters differentially expressed in shoot and root

According to Renau-Morata et al. (2021), the different responses between tissues underlined the different roles displayed by transporters to cope with low N conditions (**Tables S4**, **S5**). Transcriptomic analysis identified several N transporter genes in both tissues, which played a key role in root uptake, root to shoot and leaf to sink transport, remobilization, and storage (Tegeder and Masclaux-Daubresse, 2018), whose engineering modification improved yield or NUE (Melino et al., 2022). In our condition, two members of the high-affinity nitrate transporter NRT2 were differentially expressed between genotypes in the shoot and root. In particular, *NRT2.3* was strongly downregulated in the shoot of UC82 compared to RO, mainly after 24h LN resupply. Interestingly, these NO_3_^-^ transporters were reported to play a key role in long-distance nitrate transport from root to shoot, mainly at low external nitrate supply in rice and tomato (Tang et al., 2012; Abenavoli et al., 2016). Furthermore, RO showed significant *SlNRT2.4* N transporter downregulation in the root. Recently, a substantial overexpression of the orthologous *BnNRT2.4* was also identified in rapeseed root, which is not effective for boosting N absorption but mainly contributes to loading NO_3_^-^ in shoot phloem vessels (Kiba et al., 2012; Tong et al., 2020). Taking together, this evidence suggests that the higher N-use efficiency of RO compared to UC82 could be due to its ability to uptake nitrate by a low *NRT2.4* expression in the root and to transport NO_3_^-^ from the root to the shoot by a higher *NRT2.3* expression in the shoot.

In addition, in our experiment, 30 NPF transporters were alternatively up and downregulated in response to N resupply. *NPF3.1* was significantly upregulated in RO after 24 h LN resupply, although its expression decreased at 8 h. Interestingly, *NPF3.1* encodes for efflux-type NO_3_^-^ transporters, loading it into chloroplast stroma during NO_3_^-^ assimilation, an important physiological process in plant N nutrition and efficiency (Sugiura et al., 2007). More recently, *NPF3.1* expression was upregulated by low exogenous NO_3_^-^ concentrations and involved in GA transport in plants under low NO_3_^-^ supply (David et al., 2016). More interestingly, we detected two N transporters (included in group 5) highly upregulated in RO compared to UC82, orthologues to *NPF1.2* and *NPF8.3* in *Arabidopsis*. *NPF1.2*, classified as a low-affinity nitrate transporter, is involved in xylem-to-phloem transfer for redistributing NO_3_^-^ into developing leaves in *Arabidopsis*, a critical step for optimal plant growth performance (Hsu and Tsay, 2013). Besides, *NPF8.3* was reported to encode a di- and tri-peptide transporter able to recognize a variety of different amino acid combinations (Komarova et al., 2008), and more recently it was included among the transporters differentially expressed in rapeseed under nitrogen deficiency (Chao et al., 2021).

In the early response to N resupply, 23 N-transporters were also identified as differentially expressed in the root. Among these, group 4 that included two *NPF7.3* low affinity bidirectional NO_3_^-^ transporters, involved in root nitrate allocation but not essential for root to shoot translocation in *Arabidopsis*, was significantly upregulated in UC82 (Lin et al., 2008; Chen et al., 2012). These results might confirm a higher N-use efficiency of RO compared to UC82 due to the higher N-utilization efficiency (NUtE), which is similar between genotypes. Overall, we firstly demonstrated a significant genetic distance between RO and UC82 by SNP analysis (Tranchida-Lombardo et al., 2019), then the differences in NUE between the same genotypes were reported (Aci et al., 2021), and here, among the most significant DEGs between NUE contrasting genotypes, we identified putative genes and pathways involved in the early response to low N.

### Transcription factors

Transcription factors (TFs), which usually represent around 6% of coding sequences within a plant genome, are important regulators of plant signal transduction pathways under plant nutritional stress (Canales et al., 2014; Hoang et al., 2017). Among them, many TF families such as MYB, bHLH, bZIP, DOF, ERF, FAR1, GLK, NF-YA, NF-YB, and LOB were reported to be involved in plant N deficiency responses (Hao et al., 2011; Goel et al., 2018; Subudhi et al., 2020) and in coordination of nitrogen metabolism enzymes regulation (Zhang J. et al., 2020; Zhang X. et al., 2021). In our analysis, TFs belonging to the basic helix–loop–helix (*bHLH*) and Golden2-like (*GLK*) families were upregulated in RO shoots in early response to LN resupply (**Figure 3C**). Interestingly, *GLK2* appeared involved in the regulation of chloroplast development as well as the activation of many genes encoding chloroplast-localized or photosynthesis-related proteins, including those for chlorophyll biosynthesis, light harvesting, and electron transport (Kobayashi et al., 2013; Nguyen et al., 2014). More recently, *GLK2* overexpression was able to increase photosynthetic capacity, leading to higher biomass and grain yield in rice (Li et al., 2020). In agreement, the significant *GLK2* upregulation in RO (shoot) could determine an increase in its biomass production at LN, conferring NUE efficiency to this genotype compared to UC82.

Many other TFs were found differentially expressed in the root of RO compared to UC82 at LN, including *LOB/LBD*, *NF-YA*, *NF-YB*, *ARF*, *FAR1*, and *HSF*. The Lateral Organ Boundaries Domain TFs (*LBD/LOB37/38/39*) resulted in upregulation by NO_3_^-^ and, NO_3_^-^ a lesser extent, ${\text{NH}}_{4}^{+}$ , but they are also involved in *NIA1*, *NIA2*, and other NO_3_^-^-inducible gene downregulation (Rubin et al., 2009; Medici and Krouk, 2014). In our experiment, *LOB37* transcripts were less abundant in RO root after 24 h LN resupply, suggesting a lower repression of NO_3_^-^ assimilation-related genes compared to UC82. Finally, *NF-YA6* and *B5*, belonging to the *NF-YA* and *NF-YB* TF families, involved in many plant processes such as N nutrition and primary root growth (Ballif et al., 2011; Sorin et al., 2014), were found upregulated in RO root after 24 h LN resupply. Similarly, Renau-Morata et al. (2021) found two nuclear factors, *NF-YA5* and *9*, differentially expressed in tomato roots under N deficiency.

Overall, the results indicated a tissue-specific TF role in tomato (root), suggesting that different networks could contribute, at different tissue-scales, to cope with N limited conditions.

### Co-expression network analysis reveals N responsive modules

Nitrate regulates more than one thousand genes in both root and shoot; thus, the complex mechanisms by which NO_3_^-^ elicits changes in transcript abundance are still not fully understood (Vidal et al., 2015). Our co-expression network analysis allowed us to identify NO_3_^-^ responsive modules significantly upregulated (gray60 and magenta in root as well as brown and magenta in shoot) in response to N in both genotypes and, together with the results obtained by the STEM analysis, to highlight four main patterns of induction in response to N, interestingly highly similar in both tissues (**Figures 4A, B**). However, several modules also showed significant differences between genotypes. In particular, the N-use efficient genotype RO showed significantly higher upregulation of the gray60 module genes and significantly lower downregulation of those in the magenta module in the root. Interestingly, many hub genes in these modules might play a key role in tomato N responses. The midnight blue module included a cytokinin riboside 5’-monophosphate phosphoribohydrolase (*LOG8*, Solyc06g075090.3), an activator of cytokinin biosynthesis directly involved in nitrate signaling and N-metabolism regulation (Ruffel et al., 2011; Naulin et al., 2020). In the same module, the *ERF2* TF (Solyc01g090340.2), belonging to the AP2/ERF gene family in tomato and a homolog of the cytokinin response factor 5 encoding gene (*CRF5*), was also identified (**Figure 5A**). Interestingly, the analysis of tomato knockout mutants revealed that *CRF5* regulates leaf and flower development, appearing upregulated in response to cytokinins; these findings indicate that *SlCRF3* and *SlCRF5* are potential regulators and are involved in the regulation of tomato developmental processes associated with cytokinin or abiotic stresses (Gupta and Rashotte, 2014). These results might suggest an important regulatory role played by cytokinins in the early N differential response between N-contrasting tomato genotypes. N resupply seems to downregulate these genes after 8 h, with their expression increasing again after 24 h, mainly in RO. Moreover, an asparagine synthetase (*ASNS*, Solyc01g079880.3) upregulated in RO *vs*. UC82 was identified as a hub gene in the same module. The *ASNS* is a key enzyme in the N-metabolism involved in the hydrolyzation of glutamine to synthesize asparagine, the amino acid with the highest N/C ratio, used as the main stored and transported N form through the vascular tissues in many plants (Lea et al., 2007; Gaufichon et al., 2010; Gaufichon et al., 2013). The *ASNS* overexpression in *Arabidopsis* revealed a higher asparagine level in plant tissues together with an increased tolerance to N-deprivation (Lam et al., 2003; Igarashi et al., 2009), suggesting that this may be a good and viable strategy for improving NUE. Accordingly, our results suggested that RO showed a faster induction of these genes after initial downregulation compared to UC82, allowing RO to better withstand N-deficiency.

In the root, both the gray60 and magenta modules included many hub genes involved in N-related regulatory pathways (**Figure 5B**). In the magenta module, two nitrate transporters were detected as hub genes: *SlNRT2.4*, a high-affinity nitrate transporter homologue of *AtNRT2.4*, and *SlNPF22 (NRT1/PTR)*. Although *SlNRT2.4* expression in tomato is the least abundant among the NRT2 genes in almost all tissues, it is involved in both root and shoot NO_3_^-^ transport under N starvation in *Arabidopsis* (Kiba et al., 2012; Akbudak et al., 2022). Interestingly, its homolog in *Camellia sinensis* was detected as a hub gene among the LN responsive genes and was suggested as one of the main control factors for N uptake modulation in tea plants under low N (Zhang F. et al., 2020; Zhang F. et al., 2021). Our results indicated that, after initial induction, *SlNRT2.4* was downregulated, allowing tomato plants to maintain higher N uptake, especially under N-limited conditions.

Nitrate is also known to induce the expression of aquaporin genes in tomato, and some PIP genes were found to be correlated to NRT2 gene activity in *Arabidopsis* (Wang et al., 2001; Li et al., 2016). In our experiment, two aquaporins (*TIP2* and *PIP2*) as hub genes in the gray60 module were detected, confirming their central role in the short-term response to NO_3_^-^. The high correlation between NO_3_^-^ uptake and the hydraulic response in the root system was previously described in several plants (Górska et al., 2010), further suggesting that the differences in NUE performances between RO and UC82 might also be derived from a different regulation of genes involved in water transport. A genotype-specific hydraulic response to NO_3_^-^, putatively derived from different aquaporin protein levels, was recently detected in maize roots (Pou et al., 2022).

## Conclusion

To our knowledge, this is the first comparative transcriptomic study of two NUE-contrasting genotypes providing deep information on the early responses to NO_3_^-^ deficiency in tomato. The experimental setup allowed us to uncover some mechanisms underlying low NO_3_^-^ regulation in the high-NUE genotype, Regina Ostuni (RO). The comparative analysis revealed that most transcriptomic changes induced by N-stress occurred in the root and shoot, suggesting coordinated regulation of multiple genes and pathways between both tissues. In the root, the upregulation of the “phenylpropanoid and flavonoid biosynthesis” pathways in Regina Ostuni suggested its higher ability to enhance NO_3_^-^ deficiency tolerance compared to UC82. In the shoot, plant hormones and protein kinases signaling seemed to be involved in high NUE, providing novel insights in their interactions with NO_3_^-^, until now unexplored in tomatoes. Interestingly, several NO_3_^-^ transporters differentially expressed between genotypes were also identified in the N-use efficient genotype RO, confirming its higher ability to transport nitrate from root to shoot (long-distance) by higher *NRT2.3* and *NPF8.3* expressions. Finally, WGCNA decoded the dynamic regulatory network related to low N resupply, highlighting the key role played by cytokinins and ROS balancing in NO_3_^-^ deficiency regulation mechanisms adopted by the high-NUE genotype Regina Ostuni. The results obtained in this study represent new insights into the comprehensive understanding of genotypic differences in NO_3_^-^ regulation, utilization, and deficiency in tomatoes.

## Data availability statement

The datasets presented in this study can be found in online repositories. The names of the repository/repositories and accession number(s) can be found below: https://www.ncbi.nlm.nih.gov/, PRJNA912659.

## Author contributions

FS and MRA contributed to the conception and design of the study. MMA, AM and CC carried out the hydroponic experiments. MMA and GP organized the dataset derived from RNAseq analysis. MMA, GP and FM performed all the statistical analysis. MMA wrote the first draft of the manuscript. AM and GP wrote some sections of the manuscript. FS, GP, FM MRA contributed to the manuscript revision. All the Authors contributed to the article and approved the submitted version.
