# Supplementary material for: Short-term transcriptomic analysis at organ scale reveals candidate genes involved in low N responses in NUE-contrasting tomato genotypes
Source: Front Plant Sci. 2023 Mar 3;14:1125378. doi: 10.3389/fpls.2023.1125378 (PMC10020590; doi:10.3389/fpls.2023.1125378)
Supplement: Supplementary file 1 [file DataSheet_1.docx]

**Short-term transcriptomic analysis at organ scale reveals candidate genes involved in low N responses in NUE-contrasting tomato genotypes**

Francesco Sunseri^1,3*^, Meriem Miyassa Aci^1^, Antonio Mauceri^1^, Ciro Caldiero^1^, Guglielmo Puccio^2^, Francesco Mercati^3^, Maria Rosa Abenavoli^1^

^1^ Dipartimento Agraria, Università Mediterranea di Reggio Calabria, Reggio Calabria, Italy.

^2^ Dipartimento di Scienze Agrarie, Alimentari e Forestali, Università degli Studi di Palermo, Viale delle Scienze, 90128 Palermo, Italy.

^3^ National Research Council of Italy, Institute of Biosciences and Bioresources (CNR-IBBR), Palermo, Italy.

*** Correspondence:** Corresponding Author [francesco.sunseri@unirc.it](mailto:francesco.sunseri@unirc.it)

**
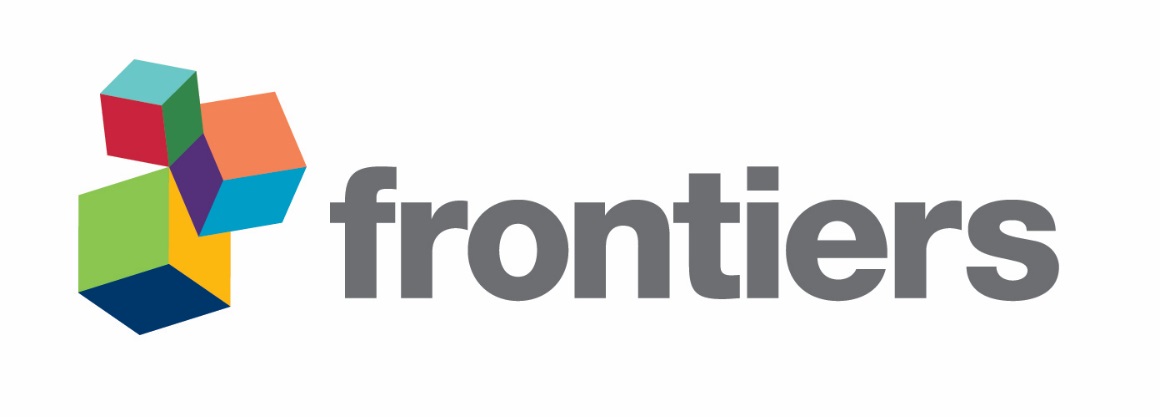
**


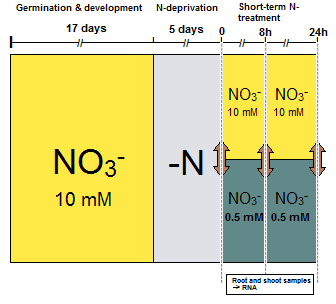


Figure S1. Experimental setup adopted for the short-term RNAseq analysis.


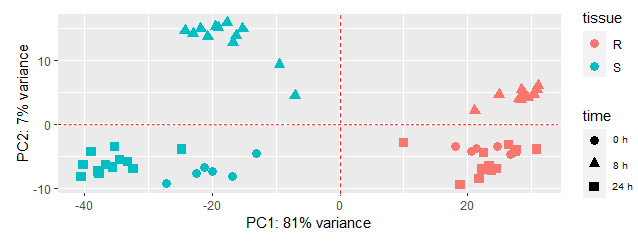


Figure S2. Principal Component Analysis (PCA) of the whole transcriptome data set. R: root; S: shoot.


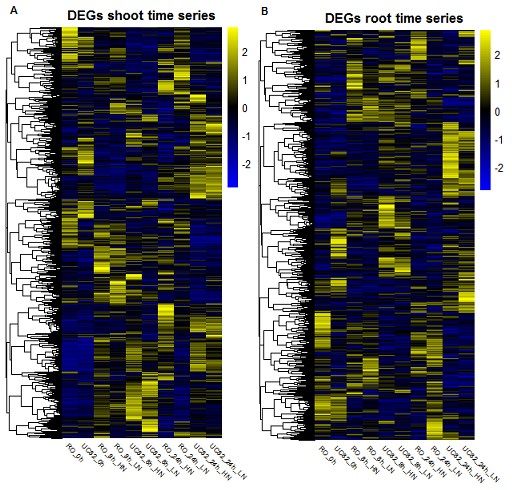


Figure S3. Hierarchical clustering of normalized expression levels of the differentially expressed genes (DEGs) between genotypes, times, N levels and their interactions.


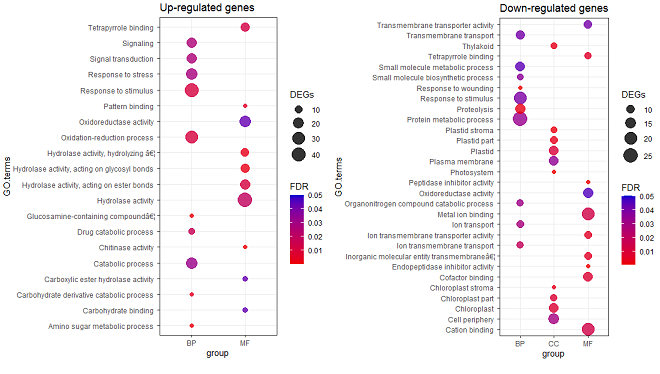


Figure S4. GO term enrichment analysis of the DEGs between RO and UC82 in shoot for the three main GO categories Biological Process (BP), Molecular Function (MF) and Cellular Component (CC).


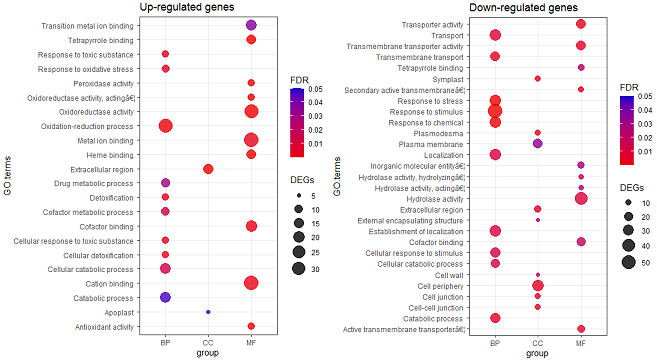


Figure S5. GO term enrichment analysis of the DEGs between RO and UC82 in root, for the three main GO categories: Biological Process (BP), Molecular Function (MF) and Cellular Component (CC).


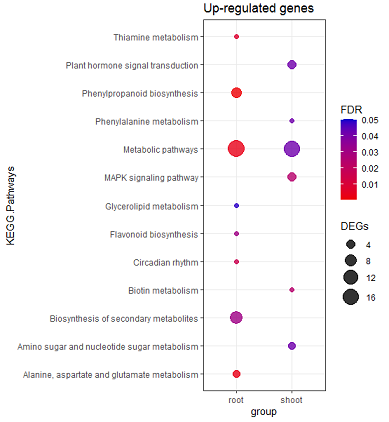


Figure S6. The KEGG pathway enrichment analysis of the up-regulated genes in RO*vs.*UC82 in both tissues.


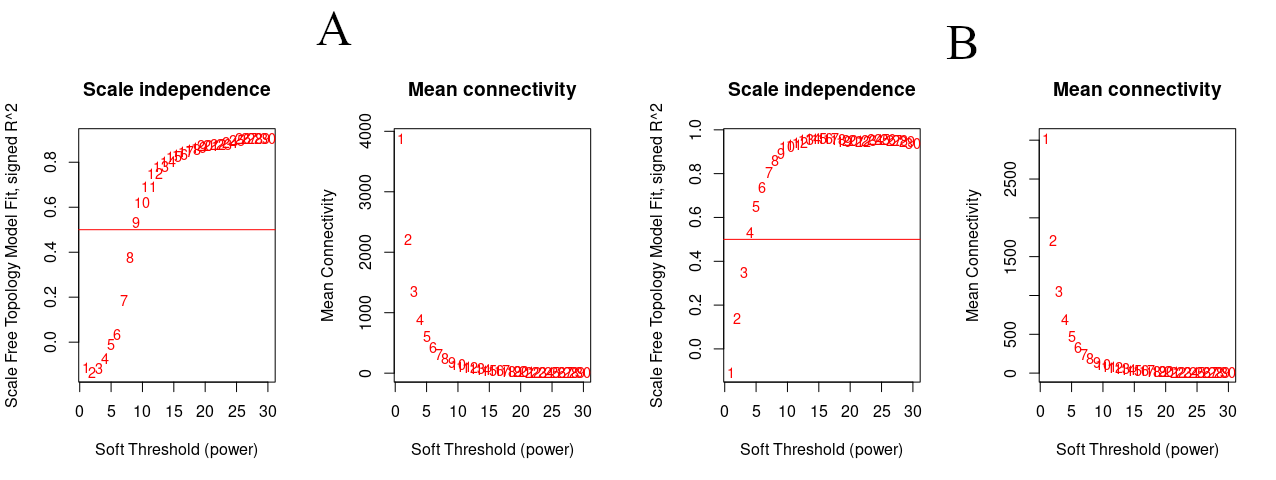


Figure S7. WGCNA Scale independence and Mean Connectivity analyses for shoot (A) and root (B) tissues.


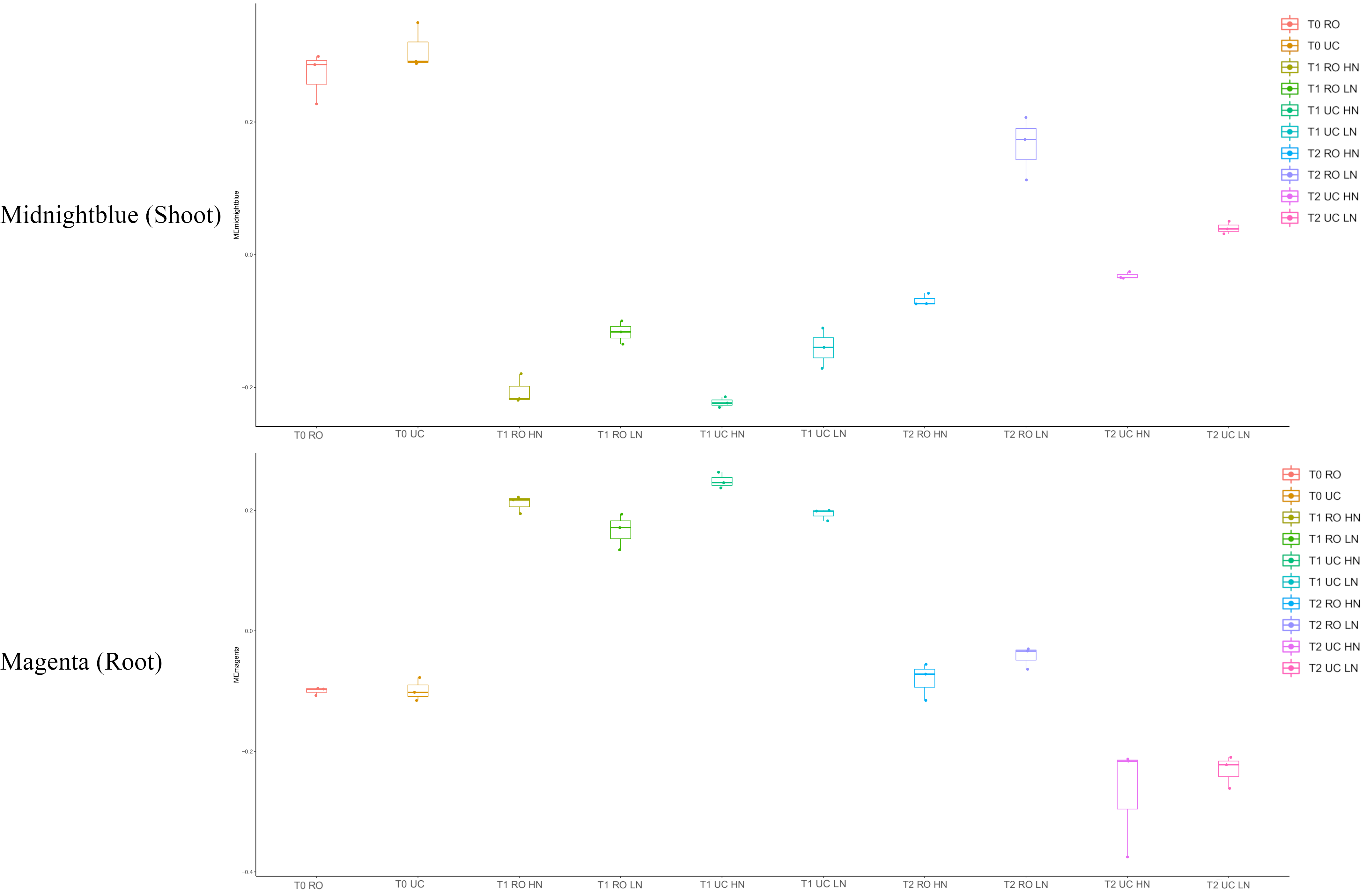


Figure S8. Box plot of Module Eigengenes for the midnightblue module (Shoot) and the Magenta module (Root).


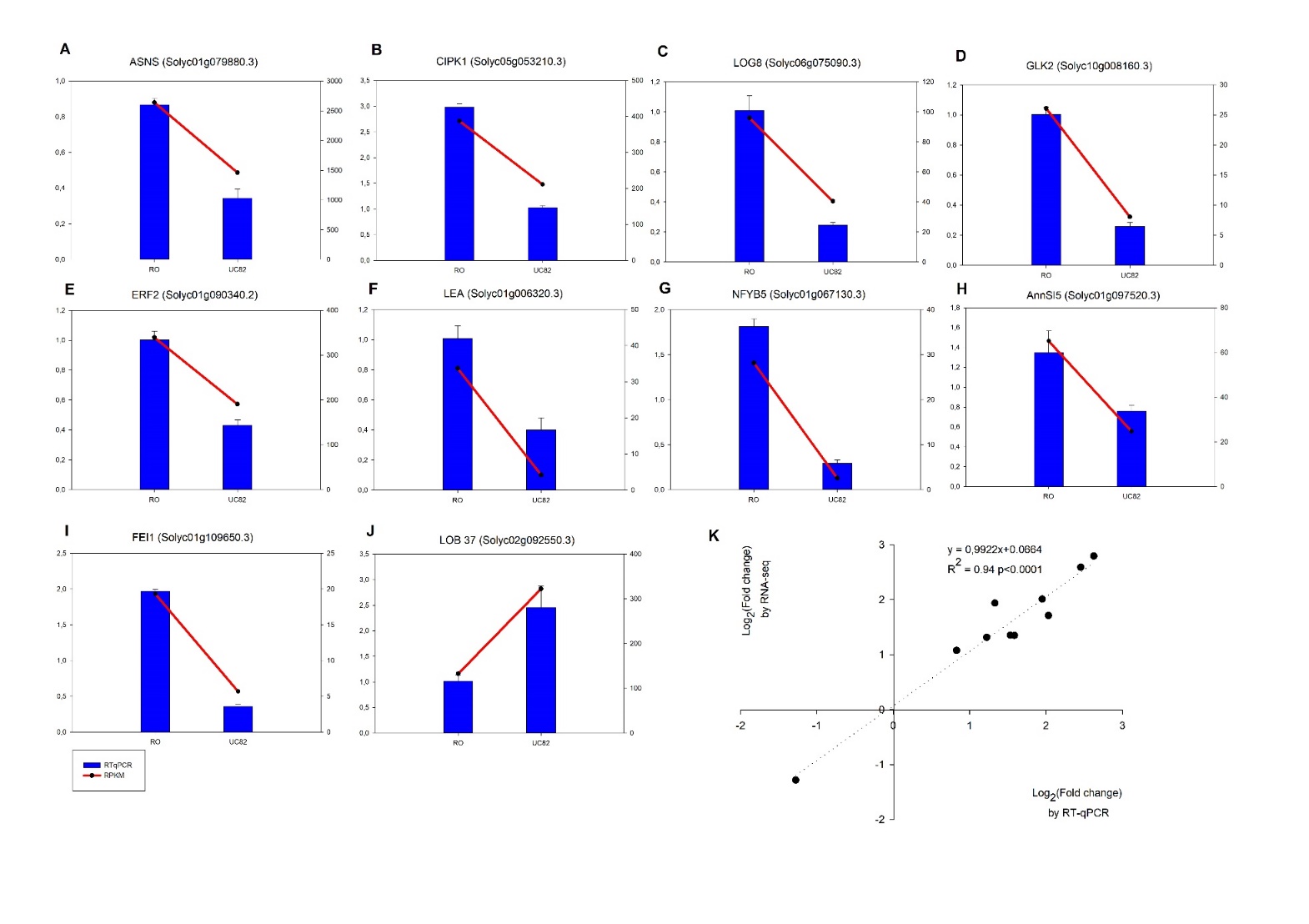


Figure S9. Confirmation of transcript levels of the differentially expressed genes (DEGs) in RO and UC82. (A-J) Transcript levels of the ten selected DEGs analysed by RT-qPCR (bars) and RNA-seq (RPKM) (red lines) in short-term analysis (24h-LN). RT-qPCR data are means ± SE (n = 3). (K) Scatter plot showing the correlation between the expression analysis by RT-qPCR (X-axis) and RNA-seq data (RPKM) (Y-axis). The relative genes expression (Ct) were normalized to the reference genes, Actin1 and Ef1-α (Lovdal and Lillo, 2009) and it was calculated for each gene by using the 2−^ΔΔCt^ method as described by Livak and Schmittgen (2001).
